# Supplementary material for: Pan-Genome Analysis of Staphylococcus aureus Reveals Key Factors Influencing Genomic Plasticity
Source: Microbiol Spectr. 2022 Nov 1;10(6):e03117-22. doi: 10.1128/spectrum.03117-22 (PMC9769869; doi:10.1128/spectrum.03117-22)
Supplement: Supplemental file 1 — Fig. S1 to S3. Download spectrum.03117-22-s0001.pdf, PDF file, 5.5 MB [file spectrum.03117-22-s0001.pdf]

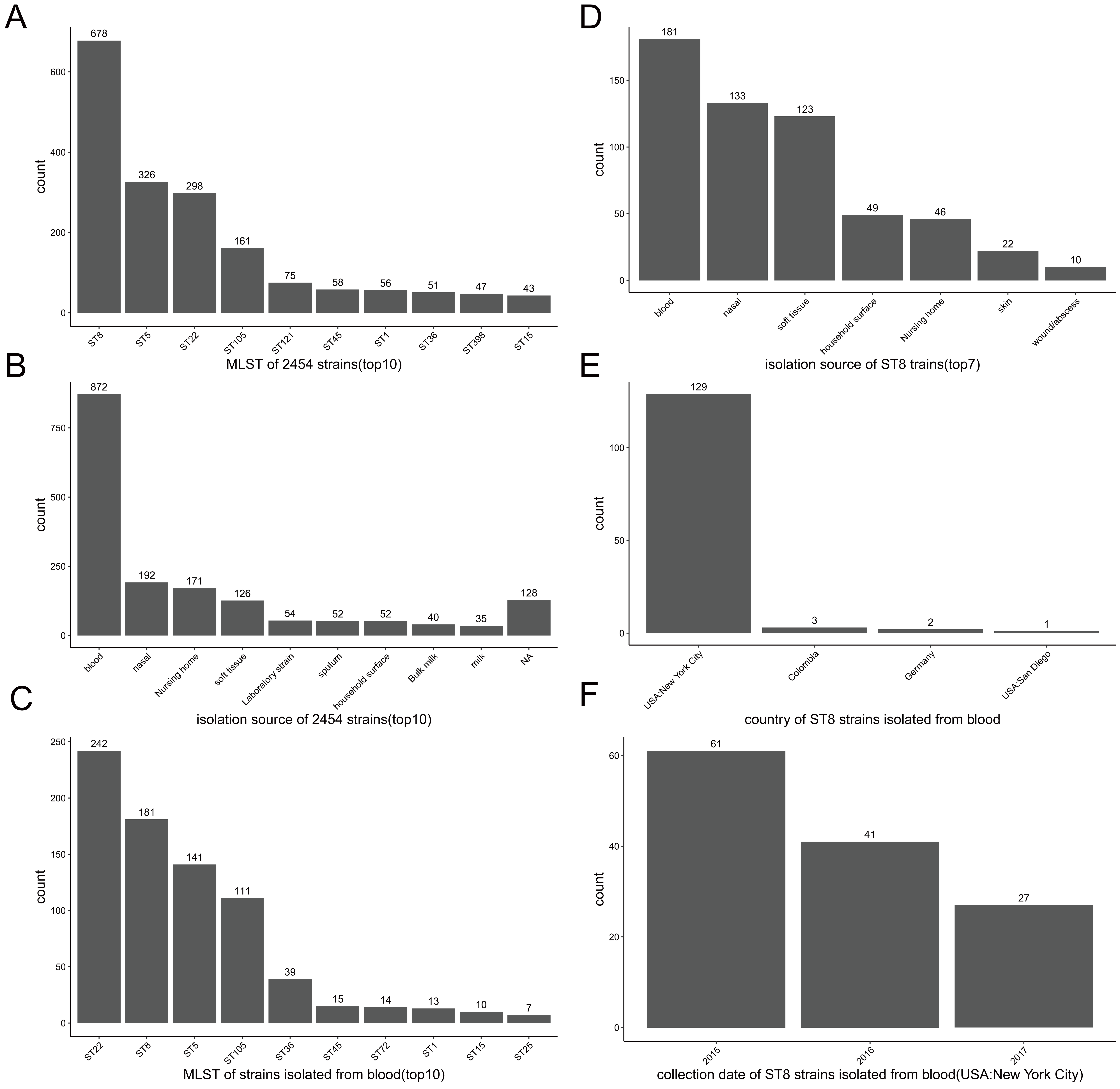

FIG S1 Statistical information of 2454 strains. The top three strains in A were selected as a group (different ST); the top three strains in C were selected as a group (different ST and isolated from blood); the top three strains in D were selected as a group (ST8 strains isolated from different sources); and the strains in F (50 strains in 2015) were selected as a group (ST8 strains isolated from blood in different years).

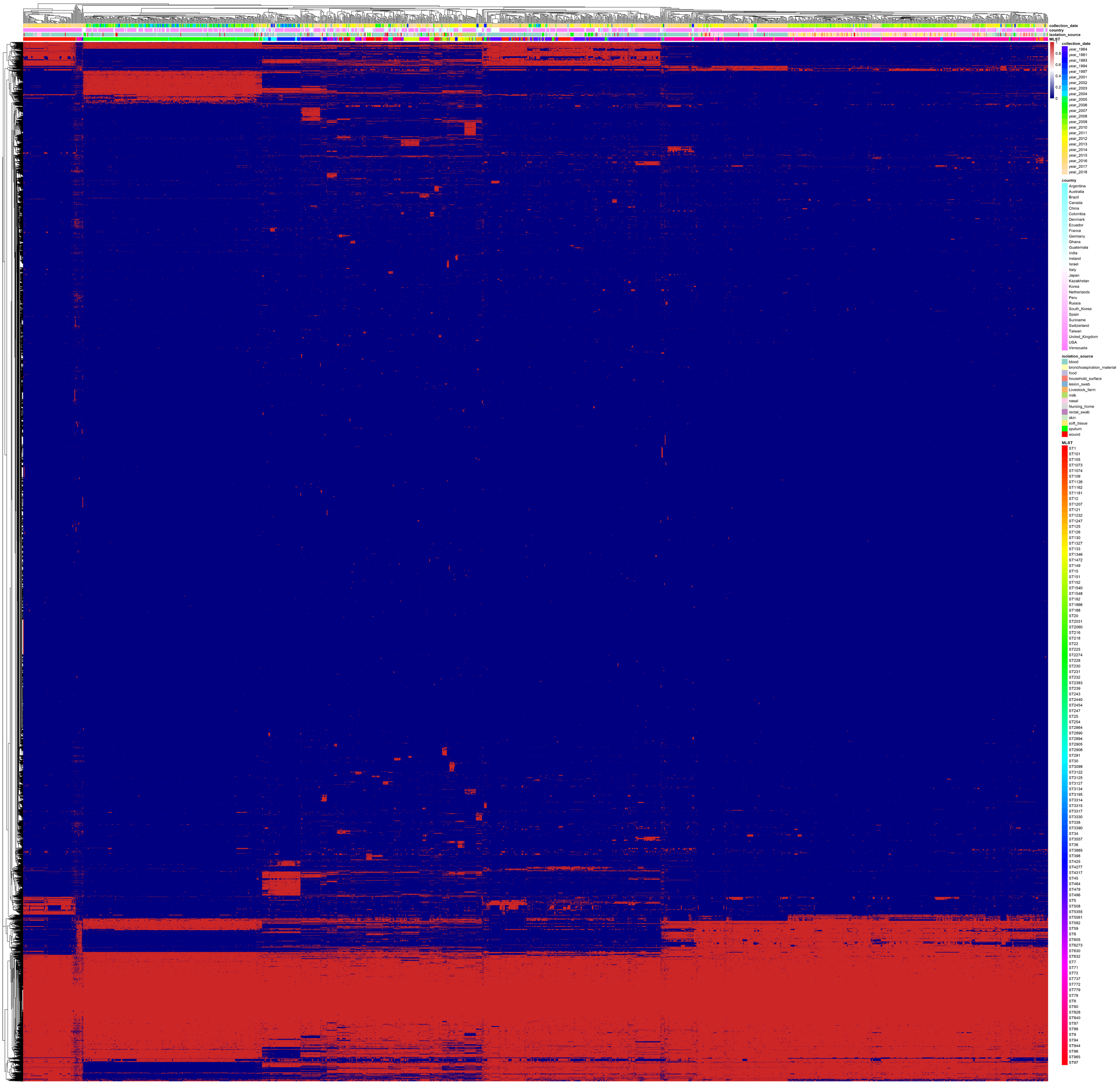

FIG S2 Heatmap of the gene-strain matrix. All genes in the 1519 *S. aureus* strains were clustered using hierarchical clustering. Strains with the same ST were clustered together and “local core gene regions” emerged.

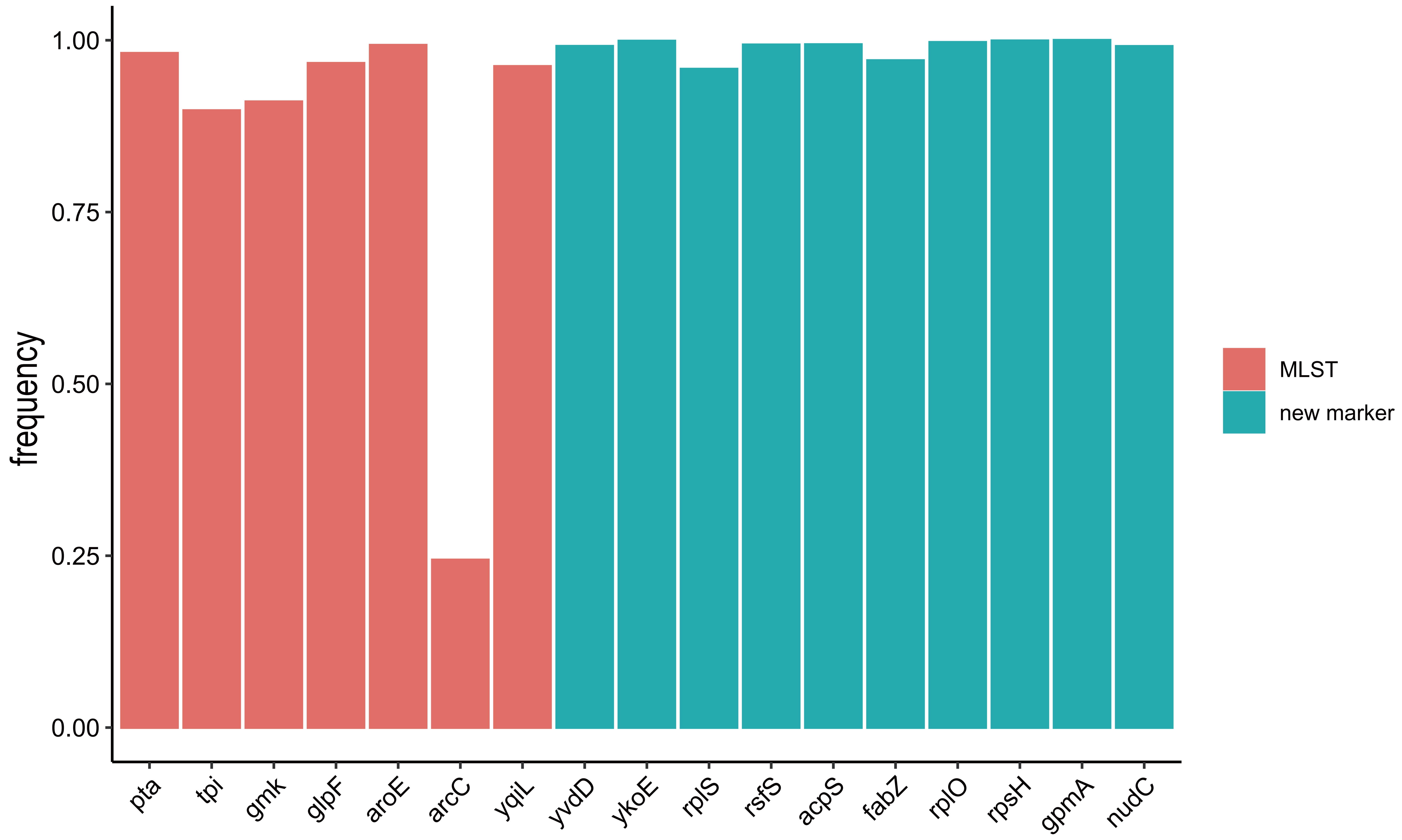

FIG S3 Frequency of carrying 7 housekeeping genes and 10 new marker genes in 5289 strains of *S. aureus*.
